# Underwater hearing in sea ducks with applications for reducing gillnet bycatch through acoustic deterrence

**DOI:** 10.1242/jeb.243953

**Published:** 2022-10-28

**Authors:** Kathleen A. McGrew, Sarah E. Crowell, Jonathan L. Fiely, Alicia M. Berlin, Glenn H. Olsen, Jennifer James, Heather Hopkins, Christopher K. Williams

**Affiliations:** ^1^ Virginia Maryland College of Veterinary Medicine, 205 Duck Pond Drive, Blacksburg, VA 24060, USA; ^2^ US Geological Survey, Eastern Ecological Science Center, 12100 Beech Forest Road, Laurel, MD 20708, USA; ^3^ Department of Entomology and Wildlife Ecology, University of Delaware, 531 South College Ave, Newark, DE 19716, USA; ^4^ Naval Undersea Warfare Center, Newport Division, Mission Environmental Planning, 1176 Howell St, Newport, RI 02841, USA

**Keywords:** Sound, Auditory, Seabird, Anthropogenic, Noise pollution, Psychoacoustics, Long-tailed duck, Surf scoter, Common eider

## Abstract

As diving foragers, sea ducks are vulnerable to underwater anthropogenic activity, including ships, underwater construction, seismic surveys and gillnet fisheries. Bycatch in gillnets is a contributing source of mortality for sea ducks, killing hundreds of thousands of individuals annually. We researched underwater hearing in sea duck species to increase knowledge of underwater avian acoustic sensitivity and to assist with possible development of gillnet bycatch mitigation strategies that include auditory deterrent devices. We used both psychoacoustic and electrophysiological techniques to investigate underwater duck hearing in several species including the long-tailed duck (*Clangula hyemalis*), surf scoter (*Melanitta perspicillata*) and common eider (*Somateria mollissima*). Psychoacoustic results demonstrated that all species tested share a common range of maximum auditory sensitivity of 1.0–3.0 kHz, with the long-tailed ducks and common eiders at the high end of that range (2.96 kHz), and surf scoters at the low end (1.0 kHz). In addition, our electrophysiological results from 4 surf scoters and 2 long-tailed ducks, while only tested at 0.5, 1 and 2 kHz, generally agree with the audiogram shape from our psychoacoustic testing. The results from this study are applicable to the development of effective acoustic deterrent devices or pingers in the 2–3 kHz range to deter sea ducks from anthropogenic threats.

## Teaser

**Highlighted Article:** Investigation of underwater hearing ability in multiple sea duck species using psychoacoustic and electrophysiological techniques, with potential for development of effective pinger devices applicable to conversation strategies to reduce sea duck bycatch.

## INTRODUCTION

Interactions between marine megafauna and commercial fisheries have occurred throughout recent history and continue to increase as a result of human population growth and industrialization of the fisheries industry (DeMaster et al., 2001; Gray and Kennelly, 2018; Lewison et al., 2004). Bycatch is a principal negative interaction between fisheries and marine animals, and typically refers to non-target animals that become hooked, trapped or entangled in fishing gear (Davies et al., 2009), resulting in mortality or injury (Biju Kumar and Deepthi, 2006). The gillnet fisheries industry has been identified as having the highest bycatch intensity score for air-breathing animals, including seabirds (Lewison et al., 2014). While many studies have addressed the effects of gillnet fisheries on marine mammals (Barlow and Cameron, 2003; Lewison et al., 2004; Read et al., 2006; Reeves et al., 2013), there is concern that >400,000 seabirds are being killed annually as bycatch across the Atlantic, Pacific and Baltic seas (Croxall et al., 2012; Žydelis et al., 2013). Pott and Wiedenfeld (2017) found that 228 seabird species have been recorded interacting with fishing gear, and Regular et al. (2013) hypothesized that diving birds are more vulnerable to gillnet bycatch compared with their surface-feeding counterparts.

The current preferred technique to mitigate depredation and bycatch of marine mammals in gillnets is the use of acoustic deterrent devices, or pingers, which emit relatively low-intensity tones (<150 dB re. 1 μPa at 1 m, RMS) at high or ultrasonic frequencies (>10 kHz, with some as high as 160 kHz). Pingers have been designed to emit tones outside the audible range for most fish species, which hear at lower frequencies (Popper and Fay, 1993), and alert non-target animals of fishing gear. Optimally, the alert causes non-target animals to exhibit avoidance behaviors and reduces the likelihood of entanglement (Dawson et al., 2013).

Controlled experiments in gillnet fisheries have shown that pingers can be effective in reducing bycatch of multiple marine mammal species (Kraus, 1999; Trippel et al., 1999; Culik et al., 2001; Barlow and Cameron, 2003; Burke, 2004; Gönener and Bilgin, 2009; Larsen and Krog, 2013). However, because there is little known about the underwater hearing abilities of seabirds, research is needed to evaluate whether existing pingers targeting marine mammals also would be effective for sea ducks or whether different pinger frequencies are needed. The only pinger study conducted on seabirds tested 1.5 kHz (±1 kHz) frequency pingers in Puget Sound, WA, USA, and saw a 50% reduction in common murre (*Uria aalge*) bycatch, but no significant effect on rhinoceros auklet (*Cerorhinca monocerata*) bycatch (Melvin et al., 1999).

In air, avian hearing abilities are typically restricted to frequencies below 10 kHz, with most species exhibiting peak sensitivity from 1 to 4 kHz (Beason, 2004; Köppl, 2015). Recent psychoacoustic and electrophysiological in-air hearing tests have been completed on multiple species of seabirds, including the common murre (*Uria aalge*), Atlantic puffin (*Fratercula arctica*), great cormorant (*Phalacrocorax carbo*), surf scoters (*Melanitta perspicillata*), white-winged scoters (*Melanitta deglandi*), black scoters (*Melanitta americana*), lesser scaup (*Aythya affinis*), harlequin ducks (*Histrionicus histrionicus*), ruddy ducks (*Oxyura jamaicensis*), common eiders (*Somateria mollissima*), red-throated loons (*Gavia stellata*) and northern gannets (*Morus bassanus*), which all share a common region of peak sensitivity between 1.0 and 3.0 kHz (Table 1; Crowell et al., 2015, 2016; Hansen et al., 2020; Larsen et al., 2020; Maxwell et al., 2016, 2017; Mooney et al., 2019, 2020; Therrien, 2014).

**Table 1.**
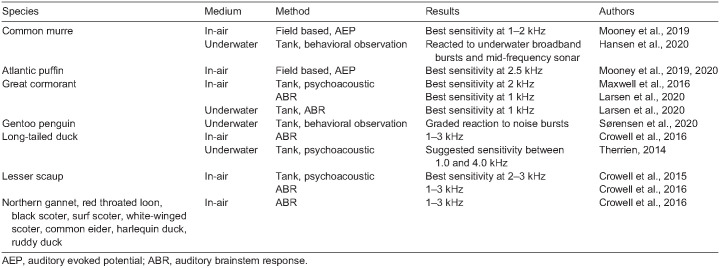
Summary of available in-air and underwater hearing data for seabird species

Underwater hearing testing is challenging to perform because of the time-consuming nature of behavioral training (for psychoacoustic testing) and the logistical efforts necessary to successfully anesthetize and intubate a bird for an underwater environment (for electrophysiological testing). Because of the inherent difficulties of testing underwater, limited data exist for underwater sensitivity on diving birds, which suggests sensitivity in the 1.0–4.0 kHz range (Table 1). Therefore, our main objective in this study was to test underwater hearing of long-tailed duck (*Clangula hyemalis*), surf scoter and common eider through the successful transfer of established methodologies used in air (both psychoacoustical and electrophysiological). Because of the logistical complications of underwater electrophysiological testing in sedated, submerged birds, we consider the results of this portion of the study preliminary, although useful. The objective for the electrophysiological portion of the study was to work through these logistical challenges and collect a limited amount of data, which should later be refined in further studies. Additionally, using baseline hearing abilities obtained through psychoacoustic techniques in the first objective, our second objective was to determine the potential efficacy of commercially available acoustic deterrent devices to avoid bycatch in gillnet fisheries.

## MATERIALS AND METHODS

Two types of auditory sensitivity investigation were used in this study: (1) psychoacoustic techniques, which involve training subjects to respond to test stimuli (e.g. pushing a lever; Dooling and Okanoya, 1995), and (2) the auditory brainstem response (ABR), which is an electrophysiological method that has been used as a tool to study the functionality of the auditory system in a wide variety of animals, including diving birds in air (Crowell et al., 2015). For both methods (psychoacoustic 2016–2018, ABR 2017–2018), ducks were tested in concrete tanks (2.5 m deep) at the US Geological Survey (USGS) Eastern Ecological Science Center's (EESC; formerly known as Patuxent Wildlife Research Center, PWRC) seabird colony in Laurel, MD, USA.

### Psychoacoustics

**Fig. 1.**
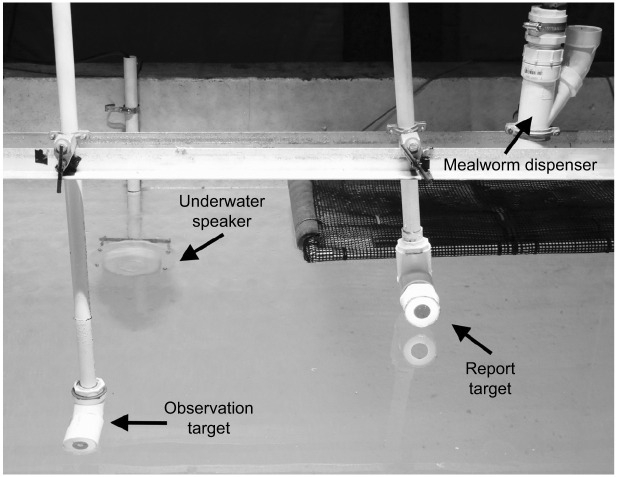
**Equipment set-up for psychoacoustic auditory testing of the three sea duck species.** Surf scoters (*Melanitta perspicillata*), long-tailed ducks (*Clangula hyemalis*), and common eider (*Somateria mollissima*) were investigated at the US Geological Survey Eastern Ecological Science Center, Laurel, Maryland, USA in 2016-2018.

In-air Go/No-go psychoacoustic procedures were described in detail in Crowell et al. (2016). For the underwater testing in this study, the observation target sat 33 cm below the surface of the water (requiring the bird to station underwater for sound playback), with the calibrated underwater speaker (University Sound UW-30, Electro-Voice, Burnsville, MN, USA) mounted on a bracket attached to the front wall of the diving tank, 30.5 cm in front of the observation target. The report target and mealworm dispenser were at the surface of the water (Fig. 1). All experimental events were coordinated by Tucker Davis Technologies TDT-RP2.1real time processer (TDT, Gainesville, FL, USA) and a desktop running specialized MATLAB code (adapted from Prior et al., 2018), which interpreted analog inputs from the ducks to a set of randomized trials to test frequency response. A hydrophone with preamplifier (Teledyne-Reson 4032, Slangerup, Denmark; sensitivity −170 dB re. 1 V μPa^−1^) was used to calibrate underwater sound stimuli. The hydrophone was positioned directly behind the observation target, at the position where the bird's ear would be located during stimulus generation. Customized MATLAB code (Edward Smith, University of Maryland, College Park, MD, USA) calibrated the speaker before each auditory sensitivity testing session and produced a set of pre-selected decibel levels within 0.05 dB of the desired level.

We trained and tested the hearing of 3 surf scoters, *Melanitta perspicillata* (Linnaeus 1758) (all males), 5 long-tailed ducks, *Clangula hyemalis* (Linnaeus 1758) (4 male, 1 female), and 1 female common eider, *Somateria mollissima* (Linnaeus 1758). Participants were raised as ducklings at the USGS EESC captive seabird facility. Ducks were housed in open-air enclosures with 3–7 ducks per pen, and given an identification based on leg band color. All research procedures were approved by the Animal Care and Use Committee at the USGS PWRC (approval #2012-06).

Experimental behavioral training and subsequent data collection trials took place from June 2016 to June 2018. We trained the ducks using operant conditioning procedures on a Go/No-go task, using mealworms as positive reinforcement (Dooling and Okanoya, 1995; Wolski et al., 2003; Skinner, 2015; Crowell et al., 2016; Maxwell et al., 2017). We began formal data collection 4–8 months after each duck's hatching date, depending on the individual bird's progress during training. To begin formal testing, ducks were required to have a hit rate of ≥80% (and a false alarm rate ≤20%) during the training phase. Because of the complicated nature of the task, we conducted intermittent training sessions in between sessions of data collection to keep the birds engaged in the task with more food opportunities.

Stimuli were presented using a modified method of constant stimuli (Dooling and Okanoya, 1995; Wolski et al., 2003; Crowell et al., 2016; Maxwell et al., 2017). The underwater auditory stimulus was a 1000 ms sinusoidal pulse with rise and fall times of 1 ms. A single pulse was chosen to accommodate the very brief period the duck remained stationary at the observation target while diving underwater. Each testing session consisted of tones played at a single frequency and a pre-selected set of seven randomly played decibel levels. For 0.5 and 4.02 kHz, the decibel levels were: 95, 105, 115, 125, 135, 135, 135 dB (re. 1 µPa). For 1.0, 2.0 and 2.96 kHz, they were: 85, 95, 105, 115, 125, 135 and 135 dB (re. 1 µPa). No tones were played >135 dB to avoid speaker distortion and hearing damage. For all frequencies, the highest decibel level (135 dB) was repeated (2 times for 1.0, 2.0 and 2.96 kHz and three times for 0.5 and 4.02 kHz). This built-in repetition ensured there would be several tones played above the individual's auditory threshold in order to provide multiple opportunities for food rewards during testing sessions. Food reward opportunities arise only if the bird can hear the tone, so three 135 dB tones were included at 0.5 and 4.2 kHz, the highest and lowest frequencies tested. Three ‘sham trials’ (control trials where no tone is played) were randomly mixed among the seven varied tones, creating a 10-trial block session. Sham trials illustrate whether the bird is following the task appropriately by exhibiting either (1) correct rejection (correctly not pecking the report target) or (2) false alarm (incorrectly pecking the report target). After a session was completed, the computer generated a new random order of the pre-selected decibel levels for the next testing session.

To standardize how hearing ability was defined across individuals, a sensory detection threshold was established. We defined this ‘threshold’ as the sound pressure level (SPL) corresponding to a 50% hit rate, as is common for psychoacoustic testing (Green and Swets, 1966). We performed a logistic regression (*P*≤0.05) on the trials of Go/No-go data across the different SPLs to ascertain the likelihood that each individual heard a specific frequency. The predicted 50% threshold for each individual at each frequency was determined from the derived logistic model. We automatically deleted (1) any 10-trial testing session with ≥2 false alarms or (2) any testing blocks or entire sessions with >20% false alarms or where any disturbance (e.g. occasional audible construction activity occurring near the building) affected the birds' normal behavior. We used univariate repeated measures analysis of variance (ANOVA) to compare thresholds across species and frequencies. Tests were considered significant at *P*≤0.05 and analyses were performed using JMP^®^ (JMP^®^ v.14.0.0, SAS Institute Inc., Cary, NC, USA, 1989-2019).

### ABR

The ABR is a scalp-recorded potential resulting from synchronized neural discharge, manifested as a series of ≥4 waves occurring within the first 10 ms following stimulation, and representing the progressive propagation of auditory neural activity through the ascending auditory pathway (Katayama, 1985; Hall, 2007; Brittan-Powell et al., 2002; Crowell et al., 2015).

ABR tests were conducted on 4 adult male surf scoters and 2 adult long-tailed ducks (1 male and 1 female) also raised from eggs at USGS EESC. Two of the 4 surf scoters had also participated in the psychoacoustic testing, and thus we were able to directly compare audiograms resulting from ABR and behavioral examinations. Prior to testing, each bird was transferred from their home pen to the building in which the psychoacoustic tests were conducted for ABR testing sessions. All ABR testing for an individual bird was completed in one session to reduce anesthesia risk.

A Lubel Labs Model 9162 acoustic source was suspended at 0.5 m depth centrally within the tank to produce stimuli. General procedures followed the in-air ABR procedures described in Crowell et al. (2015), reiterated here with differences noted. Ducks were sedated with isoflurane (Isoflurane, USP, Piramal Healthcare, Bethlehem, PA, USA) by mask (5% for induction), then intubated with a cuffed endotracheal tube and maintained on 2–4% isoflurane with oxygen at 1 l min^−1^ kg^−1^. The lowest possible percentage of isoflurane that would prevent movement in the bird was used. Isoflurane was chosen because of its history of effectiveness and safety in waterfowl (Machin, 2004; Carpenter and Marion, 2017) and Crowell et al. (2015) demonstrated that anesthesia type (isoflurane versus a ketamine/midazolam combination) does not affect thresholds in lesser scaup. Ducks were anesthetized and stabilized by the side of the tank. An esophageal stethoscope (APM Audio Patient Monitor, A. M. Bickford Inc., Wales Center, NY, USA) was used to monitor heart rate. Body temperature was monitored with a Cooper–Atkins Electro-Therm thermistor probe (Model TM99A, Middlefield, CT, USA). While in air, breathing was monitored visually.

Once the duck was sedated and intubated, it was placed inside an elastic-cotton stocking and secured via loose-fitting Velcro straps to a small platform suspended from a crane over the tank. Three standard platinum alloy needle electrodes (Grass F-Es, West Warwick, RI, USA) were placed subdermally, high on the duck's forehead (active) and directly behind each ear canal (reference and ground). Once electrodes were placed, the duck was lowered via the crane 0.5 m underwater until it was level with and facing the speaker at 1 m distance. We maintained each duck for the length of the underwater ABR trials (approximately 15 min). To counteract a natural dive reflex in submerged birds that causes a cessation of spontaneous respiration, we provided birds with assisted (manual) respiration at approximately 6 breaths min^−1^. Once the duck had been returned to the surface and the anesthetic turned off, spontaneous respiration began immediately. All ducks recovered quickly and were active and alert within 10 min of the cessation of anesthesia. No pre-operative sedation was administered, as it was possible this would interfere with the ABR, and no post-operative medication was required. Ducks remained isolated in a crate and monitored until they showed normal alertness, then were returned to their home pen and monitored there to ensure complete recovery.

Subjects were presented with stimuli made up of tone bursts of 5 ms in duration (1 ms cos2 rise/fall time and 3 ms steady-state) and 20 ms interstimulus intervals, for comparison with data from other studies (Brittan-Powell et al., 2002, 2005; Crowell et al., 2015). Tone burst frequencies were 500, 1000 and 2000 Hz, and sound pressure ranged from 105 to 145 dB re. 1 µPa. Each stimulus set was composed of a train of 9 single-frequency tone bursts that increased successively in pressure and were presented at a rate of 4 s^−1^.

Shielded electrode leads were twisted together to reduce electrical noise through common-mode rejection. Electrodes were coated in nail polish for waterproofing, leaving only the tip exposed for subdermal insertion. The stimulus presentation and ABR acquisition were synchronized using a TDT mobile real-time processor (RM2) controlled by a Gateway PC (Irvine, CA, USA). Sound stimulus waveforms were generated using OpenABR software (developed by Edward Smith, University of Maryland) and fed to the RM2 for D/A conversion, and then through an SLA-1 Studio Linear Amplifier (Applied Research and Technology, Niagara Falls, NY, USA) to drive the underwater speaker. The electrodes were connected to a TDT RA4LI headstage and RA4PA Medusa preamplifier (TDT) that amplified at 20× gain and digitized the signal before sending it over fiber optic cables to the TDT RM2, after which data were analyzed using OpenABR.

Each ABR represents the average response of 600 stimulus train presentations (alternating polarity/phase to cancel the cochlear microphonic or receptor potential generated by outer hair cells), sampled at 20 kHz for 235 ms following onset of the stimulus. This allowed for 25 ms recording time for each stimulus. The biological signal was amplified, and notch filtered at 60 Hz with the OpenABR software.

Stimulus pressures were calibrated underwater using the OpenABR software and a Cetacean Research C55 hydrophone (Cetacean Research Technology, Seattle, WA, USA) at the approximate position of the bird's head. Stimuli were calibrated with a Cetacean Research C55 model hydrophone with a sensitivity of −185 dB re. 1 V µPa^−1^ and a preamplifier gain of 20 dB. Calibration was accomplished by generating tones at a known frequency with the TDT RM2 Mobile Processor through the source at an uncalibrated target dB (typically 155 dB re. 1 µPa). This signal was then measured and interpreted with the C55 hydrophone/preamplifier setup connected to a Owon DS5032 oscilloscope (Owon, Lilliput, Zhangzhou, China) in fast Fourier transform mode to measure actual dB of signal (again re. 1 µPa) at the approximate location of the duck's head. This dB reading was fed into custom-written Matlab code to create a calibration file used by the RM2 during experimentation runs. This procedure was repeated between each individual run during the course of this project. Threshold was defined using visual detection, in which the first 10 ms of all ABR waveforms were examined visually for a response. Two independent observers were used for visual detection, and thresholds were averaged across the two observers.

## RESULTS

### Surf scoter (*Melanitta perspicillata*)

**Fig. 2.**
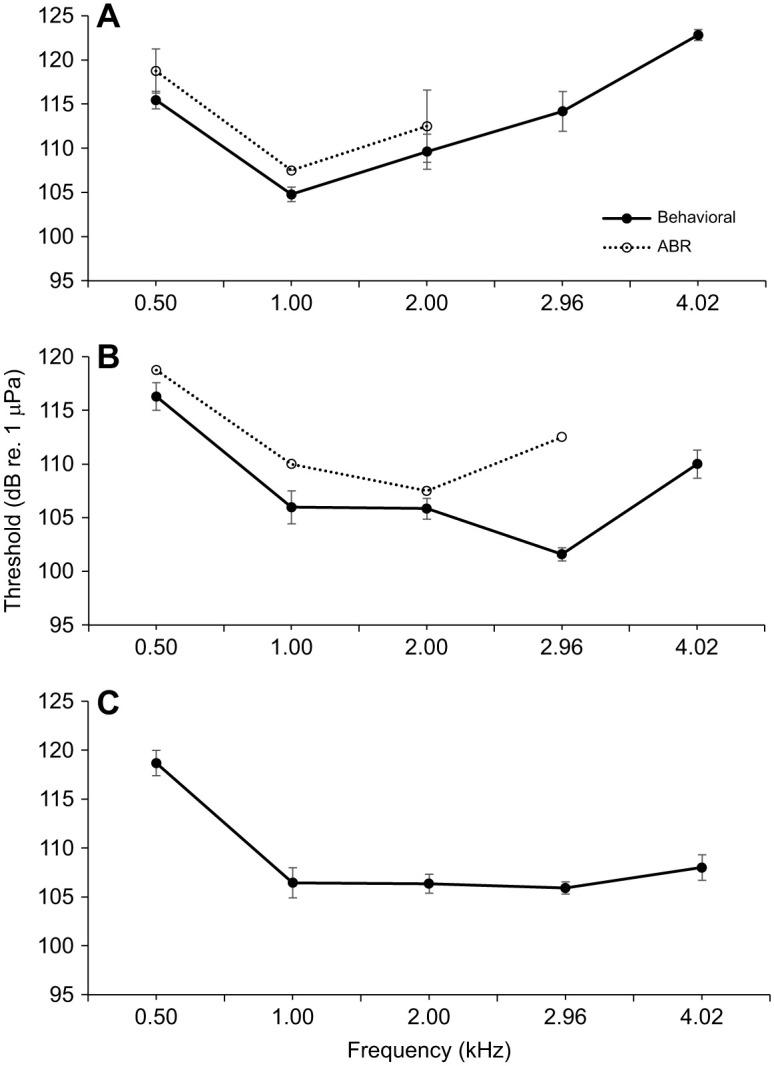
**Behavioral and auditory brainstem response (ABR) audiograms of the three duck species.** (A) Surf scoters (*Melanitta perspicillata*; *n*=3 behavioral, *n*=4 ABR), (B) long-tailed ducks (*Clangula hyemalis*, *n*=5 behavioral, *n*=4 ABR) and (C) behavioral audiogram only for a single common eider (*Somateria mollissima*) tested at the US Geological Survey Eastern Ecological Science Center, Laurel, Maryland, USA in 2017-2018. Data are means±s.d.

The psychoacoustic logistic regression models for all three individuals, frequencies and SPLs were statistically significant (χ^2^_1_≥59.46, *P*≤0.001; Table S1). The models explained 44.0–89.2% (Nagelkerke *R*^2^; Nagelkerke, 1991) of the variance in individual response to the Go/No-go task and correctly classified 79.3–88.9% of cases. The models suggested that all three surf scoters exhibited greatest sensitivity (i.e. lowest threshold) at 1.0 kHz, with a mean (±s.d.) predicted threshold of 104.8±0.8 dB (Fig. 2A). Less than 15% of sessions were discarded because of false alarm rates >20%.

ABR audiograms were obtained for four surf scoter individuals and included threshold data at three frequencies (0.50, 1.00 and 2.00 kHz; Fig. 2A). The average ABR audiogram for all four individuals was V-shaped, with peak sensitivity indicated at 1.0 kHz and a steep low-frequency roll off at 0.50 kHz. Both behavioral and ABR audiograms produced U-shaped audiograms with greatest sensitivity at 1.0 kHz.

### Long-tailed duck (*Clangula hyemalis*)

Four long-tailed ducks had complete psychoacoustic audiograms, with threshold data at 0.50, 1.00, 2.00, 2.96 and 4.02 kHz. Although incomplete, we included a fifth audiogram where 180 trials were collected at 1.0 and 2.0 kHz, 100 trials at 0.5 kHz, and no data were collected for 2.96 and 4.02 kHz. The logistic regression models for all individuals, frequencies and SPLs were statistically significant (χ^2^_1_≥40.91, *P*≤0.001; Table S1). The logistic models explained 33.0–72.9% (Nagelkerke *R*^2^) of the variance in individual response to the Go/No-go task and correctly classified 74.0–90.6% of cases. The average long-tailed duck audiogram exhibited greatest sensitivity at 2.96 kHz, with a mean (±s.d.) predicted threshold of 101.6±0.6 dB (Fig. 2B). Less than 15% of long-tailed duck sessions were discarded because of false alarm rates >20%.

ABR threshold data were collected from two long-tailed duck individuals at 1.00 kHz, and one long-tailed duck individual at 0.50, 2.00 and 2.96 kHz. The ABR audiogram of one long-tailed duck individual indicated that 2.0 kHz is the frequency of greatest sensitivity (Fig. 2B). The ABR audiogram was V-shaped and differed in the frequency of greatest sensitivity compared with the behavioral audiogram.

### Common eider (*Somateria mollissima*)

The psychoacoustic logistic regression models for all frequencies and SPLs were statistically significant for this individual (χ^2^_1_≥93.65, *P*≤0.001; Table S1). The models explained 55.2–70.7% (Nagelkerke *R*^2^) of the variance in individual response to the Go/No-go task and correctly classified 84.0–87.8% of cases. The audiogram of predicted threshold values showed a flat curve around the area of greatest sensitivity between 1.0 and 3.0 kHz (Fig. 2C). Sessions with false alarm rates >20% were discarded, resulting in 3% of session data being rejected.

## DISCUSSION

### Cross-species psychoacoustic comparison

Sensitivity for all three species was best between 1.00 and 2.96 kHz, with a steep low-frequency roll-off of approximately 10 dB per octave under 1.0 kHz (Fig. 2). However, unlike the average surf scoter audiogram, which showed a distinctive dip at 1.0 kHz, the average long-tailed duck and common eider audiograms showed similar sensitivities from 1.0 to 2.96 kHz. The average surf scoter audiogram showed the highest thresholds overall. Additionally, the average surf scoter audiogram had the steepest high-frequency roll-off of approximately 10 dB per octave above 2.0 kHz. For the behavioral hearing data, there were significant effects of frequency (*F*_4,21.5_=39.54, *P*<0.01) and the interaction of species and frequency (*F*_8,21.5_=12.71, *P*<0.01). There were no significant effects of species alone (*F*_2,5.4_=4.92, *P*=0.06). All three species exhibited similar thresholds at lower frequencies (0.5 and 1.0 kHz).

The audiograms for long-tailed ducks, surf scoters and common eider were different across frequencies, and long-tailed ducks and surf scoters showed a V-shaped audiogram, with best sensitivity between 1.00 and 2.96 kHz. Our data support previously published studies that produced underwater hearing threshold estimates in an avian species, which measured great cormorant thresholds of between 1 and 2 kHz (Hansen et al., 2017; Larsen et al., 2020). Compared with great cormorants, the duck species tested here had higher threshold levels at their most sensitive frequencies. These results may indicate that the underwater hearing abilities of sea ducks may not be as specialized as in other species of diving birds, such as great cormorants. However, both of those studies tested at a shallower depth (15 cm in Hansen et al., 2017; 10 cm in Larsen et al., 2020) compared with that used in the present study (33 cm), which may alter acoustic propagation. The acoustics of small tanks are very complex, with reflection off the walls, bottom and surface of the water affecting received sound levels for the subject (Finneran and Schlundt, 2007; Rogers et al., 2016). If electrophysiologic techniques are to be used in the future for underwater hearing testing, we recommend measuring particle motion as well as performing multiple calibrations around the circumference of the head of the bird to determine whether there is any spatial variability in sound level. Additionally, our study scope did not address the possible effects of masking from ambient environmental sound underwater or self-masking from swimming. Masking may interfere with a bird's hearing ability underwater in both a closed-tank and open-water environment (Au and Hastings, 2008) and should be considered in future studies.

One of the goals of this study was to determine the feasibility of performing electrophysiological testing underwater, and to determine whether this method of testing yields comparable results compared with psychoacoustic testing. We completed an underwater electrophysiological pilot study with the aim of validating the less time intensive of the two methods, ABR, for future testing. However, the underwater ABR results are subject to error given the uncertainty surrounding the methodology. Additionally, we must acknowledge the possible influence different acoustic environments may have on the sensitivity data between this study and others conducted at varied depths in different tank setups, particularly with regards to particle motion. To our knowledge, there is no evidence in the literature that birds detect particle motion; however, we recognize that it may be a confounding factor in our results and thus comparisons between the psychoacoustic data and ABR data should be made cautiously. Comparing psychoacoustic and ABR methodology, we found similar patterns between the two methods for surf scoters. For long-tailed ducks, ABR and psychoacoustic audiograms tracked similarly at lower frequencies but diverged at 2.96 kHz (peak psychoacoustic sensitivity was at 2.96 kHz and peak ABR sensitivity was at 2 kHz). However, only one individual was tested at 2.96 kHz, so further testing will be needed to clarify the accuracy of this greatest sensitivity. Our preliminary data show that ABR thresholds were higher than psychoacoustic thresholds at all frequencies for both species, a pattern which has been demonstrated in many other studies measuring avian thresholds (Woolley and Rubel, 1999; Brittan-Powell et al., 2002, 2005; Henry and Lucas, 2008; Crowell et al., 2016). This difference, as discussed in Crowell et al. (2016), could be attributed to several factors, including differences in stimulus characteristics and/or physiological state of the subjects. Across this study, ABR thresholds were measured at fewer frequencies because of equipment constraints and anesthesia exposure limitations, but the results do suggest that underwater ABR testing may be complementary, and possibly a viable alternative, to psychoacoustic testing, which can often take months to years to complete training and testing. To confidently validate underwater ABR as a sufficient technique for hearing testing, future studies should use step sizes smaller than ±10 dB in order to minimize error.

Including the baseline underwater hearing sensitivity data for each species in future studies would further our understanding of how these animals interact with acoustic energy in the underwater environment and how they may be affected by anthropogenic underwater noise sources. Because of the propagation efficiency of acoustic energy underwater, many aquatic animals use acoustics to communicate, navigate and detect prey (Au and Hastings, 2008). There are few data available to discern whether diving birds use sound underwater; however, there are many reasons to hypothesize that auditory cues may be important in underwater orientation, communication and/or foraging. King penguins (*Aptenodytes patagonicus*), macaroni penguins (*Eudyptes chrysolophus*) and gentoo penguins (*Pygoscelis papua*) have been found to vocalize underwater, particularly during feeding dives (Thiebault et al., 2019). Diving birds may use gradations in the underwater soundscape to locate suitable foraging areas. During the winter months, diving birds such as long-tailed ducks and surf scoters have been observed foraging on oyster reefs, which provide habitat for many important prey items, including mollusks, crustaceans and fishes (Perry et al., 2009). These populations of species that inhabit the reef generate a distinct soundscape composed of sound in the ∼2–20 kHz range that can be distinguished from lower frequencies found in adjacent soft-bottom habitats (Lillis et al., 2016). Our results suggest that sea ducks may have the ability to detect this reef soundscape, though the possible effects of masking in a marine/estuary environment raise uncertainty.

**Fig. 3.**
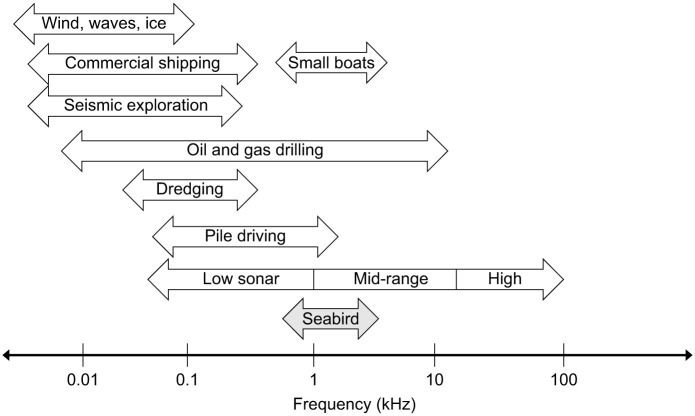
**Overlap in frequency between ambient anthropogenic noise and seabird acoustic sensitivity.** Several sources of ambient anthropogenic noise (Richardson, 1995; Bartlett and Wilson, 2002; Hildebrand, 2009) in the ocean are shown, with the measured range of greatest underwater acoustic sensitivity in the sea ducks from this study.

If seabird species do indeed use their underwater hearing abilities to locate foraging areas, anthropogenic noise in the underwater environment may interfere with this vital process. Peak underwater hearing frequencies for the three species of tested sea ducks (1–3 kHz) fell within range of several types of underwater anthropogenic noise sources, such as low sonar (<1 kHz), mid-frequency sonar (1–10 kHz), small powerboats (1–5 kHz), pile driving (0.01–1 kHz) and drilling for oil and gas (0.01–10 kHz) (Fig. 3; Richardson, 1995; Barlett and Wilson, 2002; Hildebrand, 2009). Future Ocean's ‘Net Guard’ whale pinger (Future Oceans, Buderim, QLD, Australia) emits 3 kHz tones at 145 dB, and is an example of a commercially available pinger that has the potential to effectively deter species of seabird with an underwater hearing sensitivity similar to that of long-tailed ducks and common eiders. However, this frequency is at the upper range or sensitivity for the species, and the effects of masking may be too great to provide effective deterrence. Particularly for the surf scoter, with the lowest frequency of peak sensitivity at 1.0 kHz, this unit may be ineffective. Further research should explore how grave a threat bycatch in gillnets is for surf scoters (Forsell, 1999; Regular et al., 2013) and could help to guide management decisions. Alternatively, we suggest new product development for a pinger that emits a 2 kHz tone, which has the potential to be a ‘catch-all’ for the three species tested in this study.

The scope of this study was limited to determining the baseline underwater hearing ability of three species of sea ducks and did not investigate whether an acoustic device would be effective in the goal of deterrence. Therefore, future research could first examine laboratory behavioral responses of these sea duck species to different pingers and their associated frequencies. The effects of self-masking from the bird swimming underwater should be assessed. Additionally, researchers could field-test successful lab-tested pingers in commercial gillnet fisheries (using established protocols; Dawson et al., 2013), with special consideration of unintended consequences such as drawing predators to the nets (i.e. ‘dinner bell effect’; Bordino et al., 2002) or habituation by sea ducks to the pinger device over time (Amano et al., 2017). Masking from the marine environment must be taken into consideration and potential effects on hearing thresholds should be examined. Last, we suggest that researchers expand upon previous investigations (Melvin et al., 1999; Trippel et al., 2003; Mangel et al., 2018; Cantlay et al., 2020) into visual deterrents for seabirds, particularly with species that may be poor candidates for auditory deterrence, such as surf scoters. Ultimately, a combination of methods may be the best option for deterring sea ducks.

## Supplementary Material

10.1242/jexbio.243953_sup1 Supplementary information
