# Supplementary material for: Underwater hearing in sea ducks with applications for reducing gillnet bycatch through acoustic deterrence
Source: J Exp Biol. 2022 Oct 28;225(20):jeb243953. doi: 10.1242/jeb.243953 (PMC10658911; doi:10.1242/jeb.243953)
Supplement: Supplementary information [file jexbio-225-243953-s1.pdf]

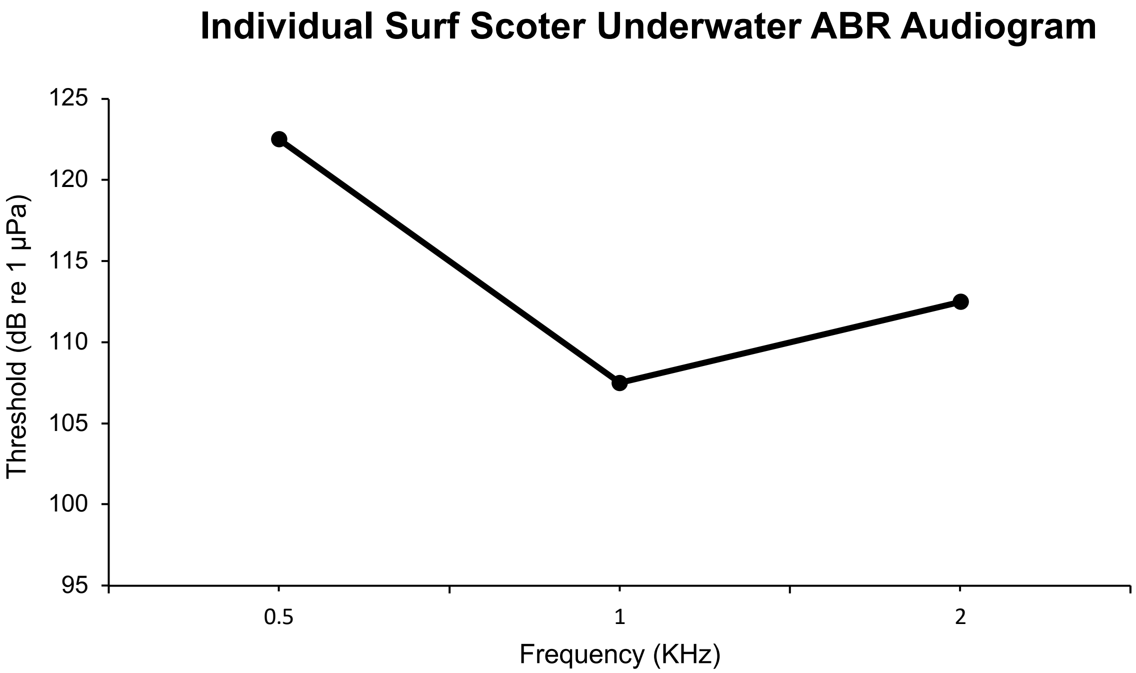

Fig. S1. An example ABR audiogram from one individual surf scoter (*Melanitta perspicillata)* tested at USGS Eastern Ecological Science Center, Laurel, Maryland, USA 2016–2018.

Table S1. Results of logistic regression performed to determine the effects of sound pressure level on the likelihood that long-tailed ducks *(Clangula hyemalis)*, surf scoters *(Melanitta perspicillata)*, and common eiders *(Somateria mollissima)* detect tones at varying frequency at USGS Eastern Ecological Science Center, Laurel, Maryland, USA 2016–2018.

Species	ID	Frequency	N trials	Nagelkerke R^2	Correctly classified cases (%)	X^2	P	Predicted 50% threshold
Long-tailed duck	White-16	0.50	150	0.63	88.0	94.96	<0.001	112.56
		1.00	180	0.73	90.6	135.72	<0.001	103.47
		2.00	180	0.68	86.7	126.97	<0.001	107.36
		2.96	180	0.65	86.1	115.46	<0.001	103.44
		4.02	150	0.52	81.3	67.81	<0.001	106.17
	Pink-16	0.50	100	0.52	81.0	49.77	<0.001	120.11
		1.00	180	0.64	85.0	118.60	<0.001	111.76
		2.00	180	0.58	78.9	102.49	<0.001	108.82
		2.96	0	-	-	-	-	-
		4.02	0	-	-	-	-	-
	Blue-17	0.50	150	0.62	85.3	91.75	<0.001	114.55
		1.00	180	0.66	89.4	117.93	<0.001	103.79
		2.00	180	0.55	84.4	92.66	<0.001	105.02
		2.96	180	0.50	82.8	76.79	<0.001	100.46
		4.02	150	0.33	76.7	40.91	<0.001	110.78
	Green-17	0.50	150	0.50	80.0	70.07	<0.001	116.84
		1.00	180	0.63	85.0	112.50	<0.001	106.41
		2.00	180	0.58	83.9	97.61	<0.001	104.18
		2.96	180	0.61	85.6	103.57	<0.001	100.63
		4.02	150	0.40	76.7	51.58	<0.001	113.25
	Orange-17	0.50	150	0.35	74.0	44.71	<0.001	117.41
		1.00	180	0.54	81.7	90.08	<0.001	104.40
		2.00	180	0.66	88.3	117.93	<0.001	103.79
		2.96	180	0.59	87.2	98.12	<0.001	101.80
		4.02	150	0.51	80.0	68.52	<0.001	109.73
	Average	0.50	700					116.29 ± SE 1.29
		1.00	900					105.97 ± SE 1.54

Journal of Experimental Biology • Supplementary information

		2.00	900					105.84 + SE 0.97
		2.96	720					101.58 ± SE 0.62
		4.02	600					109.98 ± SE 1.31
Surf Scoter	White-16	0.50	150	0.49	79.3	67.86	<0.001	116.61
		1.00	180	0.46	80.6	73.64	<0.001	106.22
		2.00	180	0.61	84.4	108.77	<0.001	112.49
		2.96	180	0.69	88.9	130.46	<0.001	116.13
		4.02	150	0.44	80.0	59.46	<0.001	124.05
	Yellow-16	0.50	150	0.62	85.3	93.19	<0.001	113.48
		1.00	180	0.63	86.1	108.85	<0.001	103.37
		2.00	180	0.62	80.0	88.31	<0.001	105.79
		2.96	180	0.71	87.8	135.02	<0.001	109.67
		4.02	150	0.58	84.0	85.45	<0.001	122.11
	Green-16	0.50	150	0.89	82.7	92.21	<0.001	116.29
		1.00	180	0.60	83.3	103.06	<0.001	104.76
		2.00	180	0.56	83.9	97.25	<0.001	110.55
		2.96	180	0.59	86.7	104.69	<0.001	116.71
		4.02	150	0.61	85.3	89.90	<0.001	122.31
	Average	0.50	450					115.46 ± SE 0.99
		1.00	540					104.78 + SE 0.82
		2.00	540					109.61 ± SE 1.99
		2.96	540					114.17 ± SE 2.26
		4.02	450					122.82 ± SE 0.61
Common Eider	White-16	0.50	150	0.71	84.0	113.33	<0.001	118.68
		1.00	180	0.55	86.1	93.99	<0.001	106.45
		2.00	180	0.55	87.8	93.65	<0.001	106.36
		2.96	180	0.62	86.1	109.10	<0.001	105.91
		4.02	150	0.67	86.7	98.46	<0.001	108.00

Journal of Experimental Biology • Supplementary information
